# Visual function and serous retinal detachment in patients with branch retinal vein occlusion and macular edema: a case series

**DOI:** 10.1186/1471-2415-11-29

**Published:** 2011-09-26

**Authors:** Hidetaka Noma, Hideharu Funatsu, Tatsuya Mimura, Katsunori Shimada

**Affiliations:** 1 Department of Ophthalmology, Yachiyo Medical Center, Tokyo Women's Medical University, Chiba, Japan; 2 Department of Ophthalmology, University of Tokyo Graduate School of Medicine, Tokyo, Japan; 3 Department of Hygiene and Public Health II, Tokyo Women's Medical University, Tokyo, Japan

## Abstract

**Background:**

The influence of serous retinal detachment (SRD) on retinal sensitivity in patients with branch retinal vein occlusion (BRVO) and macular edema remains unclear. This is despite the frequent co-existence of SRD and cystoid macular edema (CME) in BRVO patients on optical coherence tomography (OCT) and the fact that CME is the most common form of macular edema secondary to BRVO. We investigated visual function (visual acuity and macular sensitivity), macular thickness, and macular volume in patients with BRVO and macular edema.

**Methods:**

Fifty-three consecutive BRVO patients (26 women and 27 men) were divided into two groups based on optical coherence tomography findings. Macular function was documented by microperimetry, while macular thickness and volume were measured by OCT.

**Results:**

There were 15 patients with SRD and 38 patients with CME. Fourteen of the 15 patients with SRD also had CME. Visual acuity was significantly worse in the SRD group than in the CME group (P = 0.049). Also, macular thickness and macular volume within the central 4°, 10°, and 20° fields were significantly greater in the SRD group (P = 0.008, and P = 0.007, P < 0.001 and P < 0.001, and P < 0.001 and P < 0.001, respectively). However, macular sensitivity within the central 4°, 10°, and 20° fields was not significantly worse in the SRD group than in the CME group.

**Conclusions:**

SRD itself may decrease visual acuity together with CME, because nearly all SRD patients also had CME. SRD does not seem to influence macular function on microperimetry.

## Background

Branch retinal vein occlusion (BRVO) is a common retinal vascular disease that often results in macular edema, which is the most frequent cause of visual impairment in these patients[1,2]. Increased intravascular pressure and reduced blood flow in macular capillaries can lead to dysfunction of the endothelial blood-retinal barrier and to increased vascular permeability, resulting in macular edema[3]. Vascular endothelial growth factor (VEGF) has been suggested to play an important role in the pathogenesis of macular edema[4-6]. Assessment of visual acuity and measurement of foveal thickness by optical coherence tomography (OCT) are widely considered to be useful for determining the treatment strategy in patients with BRVO[7]. However, BRVO causes pathological changes (such as bleeding) that not only affect the fovea, but also the macular region and the peripheral retina, while visual acuity primarily reflects foveal function. Thus, more detailed investigation into the functional implications of anatomical and pathological changes associated with BRVO may be important.

Recently, microperimetry has been employed to assess visual function in BRVO patients with macular edema, and the retinal thickness and retinal sensitivity are reported to be related in the entire macular region affected by BRVO[8,9]. We have previously shown that retinal thickness and retinal volume are more closely associated with retinal sensitivity than with BCVA in these patients using microperimetry[10]. The influence of serous retinal detachment (SRD) on retinal sensitivity in BRVO patients with macular edema remains unclear, even though SRD and cystoid macular edema (CME) often co-exists in these patients when they are examined by OCT,[11-15] although CME is the most common form of macular edema secondary to BRVO. Some authors have reported a poor visual prognosis for BRVO patients with SRD[15-18]. Accordingly, we investigated both visual function (visual acuity and macular sensitivity) and retinal morphology (macular thickness and macular volume) to assess the influence of SRD in BRVO patients with macular edema.

## Methods

### Subjects

Informed consent to participation in this study was obtained from each subject following an explanation of the purpose and methods. This study was performed in accordance with the Helsinki Declaration of 1975 (1983 revision), and it was approved by the ethics committee of Tokyo Women's Medical University. Fifty-six consecutive patients were included in this prospective uncontrolled study conducted at the Department of Ophthalmology of Tokyo Women's Medical University between March 2008 and November 2010 (Table 1). Each patient had unilateral BRVO.

**Table 1:** Baseline Clinical Features of the CME and SRD Groups

Findings	CME (N = 38)	SRD (N = 15)	P value
Age (years)	68.2 ± 9.4^‡^	70.7 ± 10.5^‡^	0.398
Gender (female/male)	18/20	8/7	0.696
Hypertension	27	9	0.437
Systolic blood pressure (mmHg)	137 ± 15^‡^	128 ± 16^‡^	0.054
Diastolic blood pressure (mmHg)	82 ± 10^‡^	80 ± 12^‡^	0.523
Hyperlipidemia	17	6	0.754
Duration of BRVO (months)	4.4 ± 2.7^‡^	3.8 ± 1.5^‡^	0.380
Pattern of BRVO (major/macular)	24	13	0.093
Nonperfused area (disc areas)	32.6 ± 34.9^‡^	39.2 ± 37.8^‡^	0.543

BRVO = branch retinal vein occlusion; CME = cystoid macular edema; SRD = serous retinal detachment. ^‡^Mean ± standard deviation (SD).

All of the patients had CME or SRD (≥250 μm on OCT) involving the foveal center. The exclusion criteria were (1) previous ocular surgery, (2) diabetes mellitus with diabetic retinopathy, (3) previous macular laser photocoagulation, (4) previous intravitreal injection of triamcinolone acetonide or anti-VEGF agents, (5) a history of ocular inflammation, and (6) marked retinal hemorrhage (including macular bleeding involving the intrafoveal or subfoveal spaces). Patients with marked retinal hemorrhage were excluded from the study because we could not judge whether SRD or CME had an influence on retinal sensitivity, which could have altered the results of our correlation analysis. Twenty-eight patients had superior vein occlusion and 25 patients had inferior occlusion.

### Fundus Examination

At baseline screening, patients underwent ophthalmoscopy and biomicroscopic examination using a slit-lamp with a fundus contact lens. They also underwent standard fundus color photography and fluorescein angiography, which was performed with a Topcon TRC-50EX fundus camera, an image-net system (Tokyo Optical Co. Ltd., Japan), and a preset lens with a slit-lamp.

A masked grader independently assessed ischemic retinal vascular occlusion on the fluorescein angiograms by measuring the ischemic area of the retina with the public domain Scion Image program, as reported previously[4-6]. On digital photographs of the fundus, the optic disc was outlined with a cursor and then its area was measured, as was also done for the nonperfused area of the retina. Then the nonperfused area was divided by the disc area to calculate the severity of retinal ischemia.

In addition, macular sensitivity was investigated by microperimetry, and macular thickness and macular volume were measured by OCT.

### Measurement of BCVA

Each patient underwent measurement of best-corrected visual acuity (BCVA) with an SC-2000 System chart (Nidek, Gamagori, Japan). BCVA was measured in decimal units on a Landolt chart by orthoptists. The chart brightness was set at 80-320 cd/m^2 ^and chart contrast was more than 74%. The results were converted to the logarithm of the minimum angle of resolution scale (log MAR).

### Optical Coherence Tomography

OCT was performed with an instrument from Zeiss-Humphrey Ophthalmic Systems (Zeiss Stratus OCT3, Carl Zeiss Meditec, Dublin, CA, USA) to measure the foveal thickness. At each visit, all patients underwent OCT of vertical retinal cross-sections with the instrument centered on the fovea and in the fast macular thickness mode. On these views, retinal thickness was defined as the distance between the inner surface of the neurosensory retina and the retinal pigment epithelium. Foveal thickness was calculated as the average macular thickness within a circle with a radius of 500 μm centered on the fovea. A macular thickness map and macular volume map were obtained by scanning 6 × 6 mm (20° × 20°) areas of the macular region, which was divided into the following nine subfields: 1) fovea, 2) superior inner macula, 3) nasal inner macula, 4) inferior inner macula, 5) temporal inner macula, 6) superior outer macula, 7) nasal outer macula, 8) inferior outer macula, and 9) temporal outer macula[10]. The diameters of the central, inner, and outer circles were 1, 3, and 6 mm, respectively. Measurement of the retinal thickness and retinal volume in each region was automatically performed by computer software. The mean macular thickness was determined for the foveal subfield covering the central 1 × 1 mm (4° × 4°), for five subfields (fovea, superior inner, nasal inner, inferior inner, and temporal inner) covering the central 3 × 3 mm (10° × 10°), and for all nine subfields covering the entire central 6 × 6 mm (20° × 20°).

Macular volume was calculated as follows. A central macular thickness map measuring 6.00 mm in diameter was generated and this map was divided into nine quadrants. The diameters of the middle and inner circles were 3.00 mm and 1.00 mm, respectively. The mean macular thickness was calculated by the Macular Volume Protocol of the Stratus OCT for each of the nine quadrants on the radial scans, and then the mean thickness was multipled by the quadrant area to calculate the volume of each quadrant. The total macular volume for the foveal subfield covering the central 1 × 1 mm (4° × 4°), for five subfields (fovea, superior inner, nasal inner, inferior inner, and temporal inner) covering the central 3 × 3 mm (10° × 10°), and for all nine subfields covering the entire central 6 × 6 mm (20° × 20°) was thus determined as the sum of the relevant quadrant volumes.

We divided the BRVO patients into two groups depending on whether or not SRD was detected by OCT[19]. SRD was defined as typical subretinal fluid accumulation leading to detachment of the neurosensory retina with low or absent reflectivity anterior to a clearly distinguishable outer band irrespective of the presence of CME. On the other hand, CME was defined as hyporeflective intraretinal cavities on OCT.

### Functional Mapping by Microperimetry

Microperimetry was performed with the MP-1 microperimeter (Nidek, Gamagori, Japan) using an infrared fundus camera with a liquid crystal display controlled by dedicated software. The MP-1 performs microperimetry and assesses fixation independently with an automated eye tracking system that provides real-time compensation for eye movements and therefore allows presentation of a stimulus at precisely the predefined retinal location. That is, if the reference area moves, the stimulus is also automatically moved, while the stimulus is not delivered if the reference area cannot be detected. The macular sensitivity threshold can be measured easily because the strength of the stimulus is altered automatically and progressively during microperimetry. Color fundus photographs can also be acquired and the findings can be registered either automatically or manually along with the infrared image. At the end of testing, microperimetry data can also be superimposed on the digital fundus photograph. Thus, microperimetry is performed while observing a target set on the fundus, so the target is precisely located and testing is reliable even in patients who do not have stable fixation.

Microperimetry settings were identical for all examinations, and Goldmann III stimuli were presented in random order according to a 4-2-1 double staircase strategy. The stimulus intensity ranged from 0 to 20 decibels (dB) (0 dB corresponded to the strongest signal intensity of 127 cd/m^2^) in 1-dB steps, and the duration of each stimulus was 200 ms. The target was varied in size according to the patient's visual acuity. Macular sensitivity maps were obtained by using the macula 20 degrees program of the MP-1. Mean macular sensitivity was calculated from the sensitivity in each of the nine subfields on the retinal map generated by OCT[10]. The mean macular sensitivity was determined for five locations covering the central 4° field, 29 locations covering the central 10° field (five subfields: fovea, superior inner, nasal inner, inferior inner, and temporal inner), and 57 locations covering the entire central 20° field (all nine subfields).

### Statistical Analysis

All analyses were performed with SAS System 9.1 software (SAS Institute Inc., Cary, North Carolina, USA). Results are presented as the mean ± SD or as frequencies. Student's *t*-test or one-way analysis of variance was employed to compare normally distributed unpaired continuous variables between the groups. The chi-square test was used to compare nominal variables. Two-tailed P values of less than 0.05 were considered to indicate statistical significance.

## Results

The BRVO patients (26 women and 27 men) were aged 68.9 ± 9.7 years (mean ± SD). The mean duration of BRVO was 4.3 ± 2.5 months (range: 1 - 12 months). The average nonperfused area was 34.5 ± 35.6 disc areas, with a range of 0 to 117 disc areas. The characteristics of the CME and SRD groups are summarized in Table 1. Among the 53 patients with BRVO, 38 were assigned to the CME group and 15 to the SRD group. The mean age, female/male ratio, prevalence of hypertension, prevalence of hyperlipidemia, duration of BRVO, incidence of major BRVO, and nonperfused area of the retina were similar in the CME and SRD groups (*P *= 0.398, *P *= 0.696, *P *= 0.437, *P *= 0.754, *P *= 0.380, *P *= 0.093, and *P *= 0.543, respectively). Fourteen (93%) of the 15 patients in the SRD group had both SRD and CME, whereas 1 patient (7%) had SRD alone.

**Figure 1:**
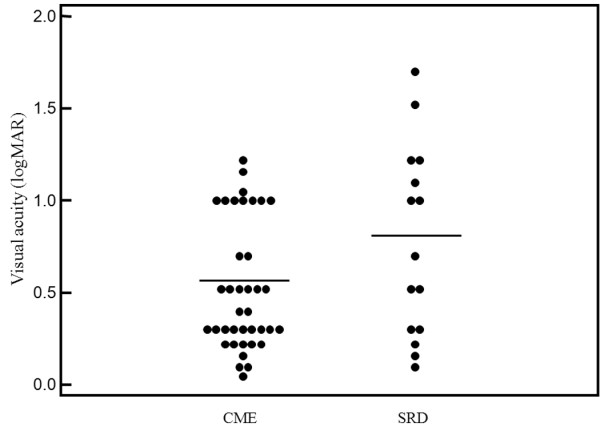
**Best-corrected visual acuity (BCVA) of the CME and SRD groups converted to the logarithm of the minimum angle of resolution scale (log MAR)**. There was a significant difference of visual acuity between the two groups (P = 0.049).

**Figure 2:**
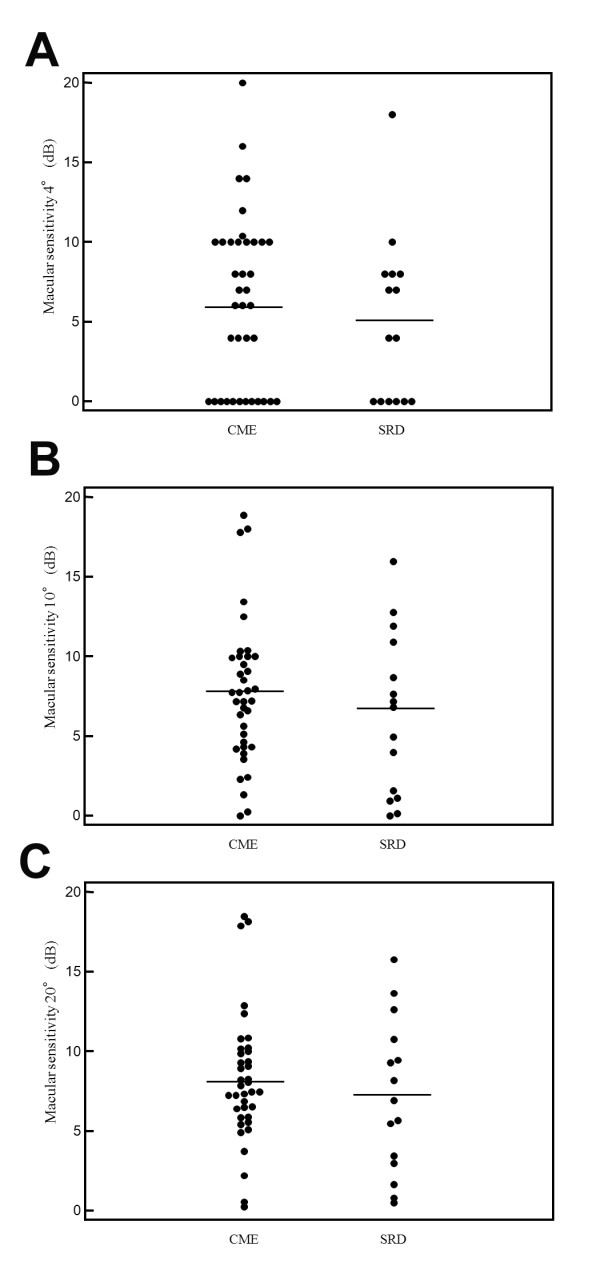
**Macular sensitivity in the CME and SRD groups**. There were no significant differences of macular sensitivity within the central (A) 4°, (B) 10°, and (C) 20° fields between the two groups (P = 0.413, P = 0.338, and P = 0.402, respectively).

**Figure 3:**
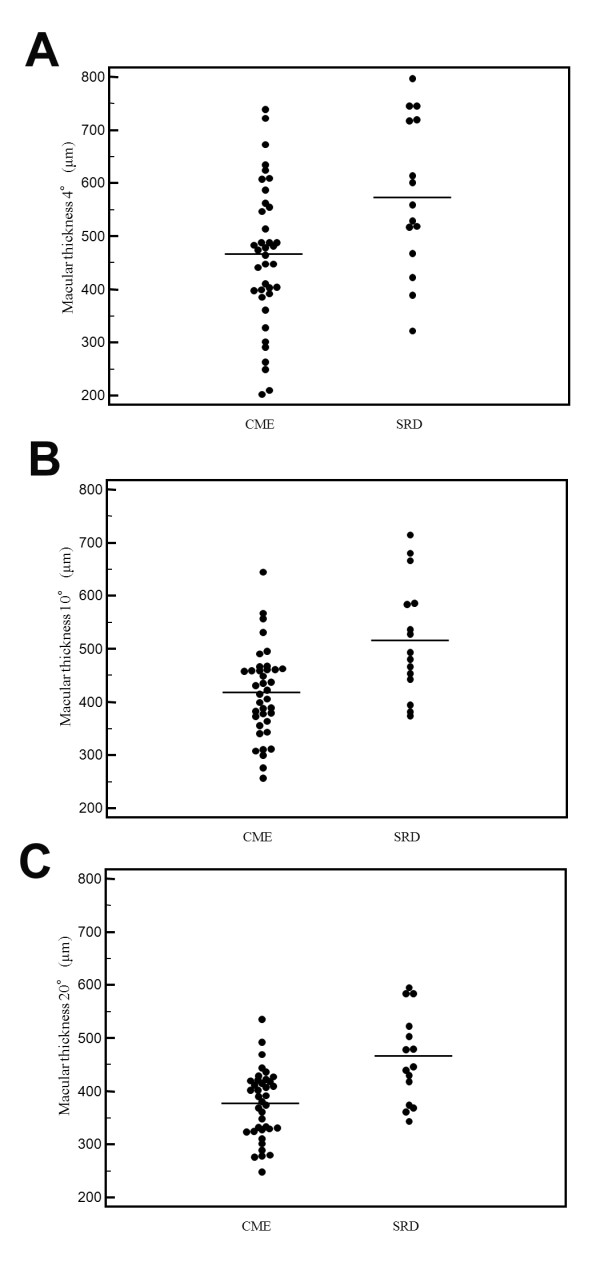
**Macular thickness in the CME and SRD groups**. There were significant differences of macular thickness within the central (A) 4°, (B) 10°, and (C) 20° fields between the two groups (P = 0.008, P < 0.001, and P < 0.001, respectively).

**Figure 4:**
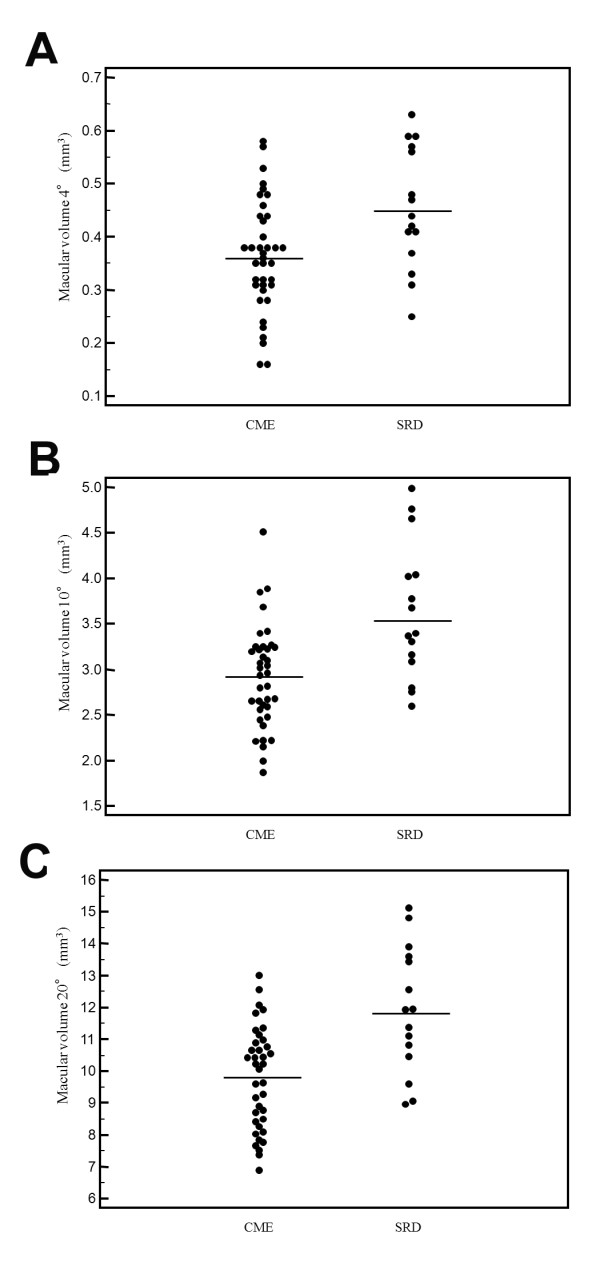
**Macular volume in the CME and SRD groups**. There were significant differences of macular volume within the central (A) 4°, (B) 10°, and (C) 20° fields between the two groups (P = 0.007, P < 0.001, and P < 0.001, respectively).

As shown in Figure 1, visual acuity was significantly worse in the SRD group than in the CME group (0.77 ± 0.52 vs. 0.52 ± 0.35, P = 0.049). However, as shown in Figures 2A-C, macular sensitivity within the central 4°, 10°, and 20° fields was not significantly worse in the SRD group than in the CME group (4.9 ± 5.2 dB, 6.3 ± 5.1 dB, and 7.1 ± 4.8 dB vs. 6.3 ± 5.4 dB, 7.7 ± 4.5 dB, and 8.2 ± 4.1 dB, respectively; P = 0.413, P = 0.338, and P = 0.402, respectively). Conversely, the macular thickness within the central 4°, 10°, and 20° fields was significantly greater in the SRD group than in the CME group (578 ± 145 μm, 519 ± 109 μm, and 462 ± 83 μm vs. 462 ± 134 μm, 417 ± 83 μm, and 375 ± 65 μm, respectively; P = 0.008, P < 0.001, and P < 0.001, respectively) (Figures 3A-C). Also, the macular volume within the central 4°, 10°, and 20° fields was significantly greater in the SRD group than in the CME group (0.46 ± 0.12 mm^3^, 3.6 ± 0.75 mm^3^, and 11.9 ± 1.98 mm^3 ^vs. 0.36 ± 0.11 mm^3^, 2.9 ± 0.56 mm^3^, and 9.8 ± 1.59 mm^3^, respectively; P = 0.007, P < 0.001, and P < 0.001, respectively) (Figures 4A-C).

## Discussion

In the RETINA study,[10] we divided the macular region into nine sections, which were (1) fovea, 2) superior inner macula, 3) nasal inner macula, 4) inferior inner macula, 5) temporal inner macula, 6) superior outer macula, 7) nasal outer macula, 8) inferior outer macula, and 9) temporal outer macula). We found that retinal sensitivity and thickness were correlated in each section, and multiple regression analysis confirmed this. In the present study, we divided the macular region into the following three sections: 1) 1 × 1 mm (4° × 4°) (corresponding to the fovea section in the RETINA study), 2) 3 × 3 mm (10° × 10°) (corresponding to the fovea, superior inner, nasal inner, inferior inner, and temporal inner sections), and 3) 6 × 6 mm (20° × 20°) (corresponding to all 9 sections). Then we investigated whether the retinal sensitivity and thickness of these 3 sections showed any differences between patients with SRD and those with CME.

We found that the mean visual acuity (log MAR) was significantly worse in the SRD group than the CME group, a result that is in agreement with previous reports,[15-18] although a lower percentage of our patients had SRD compared with the other reports[12,14,15]. The inner half of the fovea contains an inverted cone-shaped zone of Müller cells (the Müller cell cone), the apex of which is located at the external limiting membrane (ELM) of the fovea centralis. The cytoplasm of cells in this cone extends obliquely outward and forms the internal limiting membrane at the clivus of the foveal depression[20,21]. In eyes with retinal vein occlusion, leakage from the affected retinal capillaries accumulates around the fovea and causes retinal thickening, which may cause the internal limiting membrane at the clivus of the fovea to protrude with formation of foveal cystoid spaces[15]. The cytoplasm of the Müller cell cone would then extend perpendicularly in the walls of the foveal cystoid spaces. When leakage increases, further traction on the Müller cell cone would lead to traction on the inner and outer segments of the foveal photoreceptors, resulting in a small area of retinal detachment at the fovea[15]. Subsequently, SRD would occur when the ELM barrier breaks down at the fovea[15]. Loss of the ELM barrier often results in damage to foveal photoreceptors in the outer segment and impairment of visual acuity. Thus, the mean visual acuity of the SRD group may have been significantly worse because of damage to foveal photoreceptor cells due to foveal detachment. In addition, nearly all of the SRD patients in this study also had CME. Therefore, SRD itself may decrease visual acuity together with CME.

Interestingly, unlike the results for visual acuity, macular sensitivity within the central 4°, 10°, and 20° fields was not significantly worse in the SRD group than in the CME group. This suggests that SRD itself has no influence on macular sensitivity because there was no difference of sensitivity between the SRD and CME groups. Therefore, evaluation of both visual acuity and macular sensitivity may be important in BRVO patients to detect the effects of macular edema. There is evidence that the visual function of BRVO patients after treatment of macular edema is not fully described by measuring visual acuity: 1) Subjects with good visual acuity sometimes have difficulty seeing certain objects and the opposite also occurs[9]. 2) In some BRVO patients, there is no improvement of visual acuity after improvement of macular edema and SRD[22-24]. Thus, evaluation of both visual acuity and macular sensitivity may be clinically important for assessing visual function in BRVO patients with SRD. However, microperimetry may be less informative when the edema is very severe and visual acuity is poor, in which case macular sensitivity was reduced significantly in both groups.

We also found that the macular thickness within the central 4°, 10°, and 20° fields was significantly greater in the SRD group than in the CME group. Macular thickness increases along with vascular permeability, and the macular region and peripheral retina have abundant capillaries unlike the fovea, so extra-foveal BRVO could influence both retinal thickness and volume at the fovea. In BRVO patients, SRD may be caused by transudation of extracellular fluid into the subretinal space,[11-15] with the site of detachment being determined by the foveal architecture, especially the presence of the Müller cell cone[15]. In addition, when the barrier function of the ELM breaks down due to traction on the Müller cell cone, intraretinal fluid will move into the subretinal space, resulting in an increase of SRD and alleviation of retinal edema[15]. This may explain why macular thickness and volume were significantly greater in the SRD group than the CME group.

Surprisingly, macular sensitivity and the macular thickness/volume within the central 4°, 10°, and 20° fields did not show parallel changes in the present study, although we have previously reported that retinal thickness and retinal volume show a closer association with retinal sensitivity than with BCVA in BRVO patients with macular edema[10]. This may be because the influence of SRD on retinal function is weak since SRD is mainly caused by transudation of extracellular fluid into the subretinal space[11-15]. In addition, because there is little traction on the Müller cell cone in the macular region, except at the fovea,[15] the following processes 1)-4) leading to SRD may not occur in the macular region apart from at the fovea. 1) Traction on the Müller cell cone leads to traction on the inner and outer segments of the photoreceptors. 2) Traction on the photoreceptors causes breakdown of the barrier function of the ELM. 3) Loss of ELM barrier function results in damage to the outer segment photoreceptors. 4) Damage to the outer segment photoreceptors reduces macular sensitivity. Because it is unclear why macular sensitivity and macular thickness/volume did not show parallel changes in our BRVO patients with SRD, further investigation is needed to clarify the relations between macular function and morphology.

One limitation of this study is that it was cross-sectional and thus did not provide data on the response to treatment or prognosis of our patients, such as whether subjects with or without SRD had any particular outcomes. Because it has been reported that diffuse disorganization of the outer photoreceptor layer beneath the fovea often leads to poor visual acuity after complete resolution of macular edema and SRD,[22-24] it is possible that such diffuse disorganization of the outer photoreceptors could have affected visual acuity in our patients. In addition, Tsujikawa and associates[15] reported that dome-shaped retinal detachment is sometimes associated with a focal defect of the outer segment photoreceptors above the site of SRD, so the visual prognosis would be poor if such a defect involved the fovea. However, we could not assess the influence of changes at the junction between the inner and outer segments of the photoreceptor layer on the visual prognosis because detection of this junction was difficult with our OCT equipment. Moreover, small pointed retinal detachment (the initial stage of SRD [15]) may have been present in the CME group, but we could not assess such detachment with our OCT equipment. Accordingly, the prognosis of patients with BRVO and SRD needs to be investigated in more detail in the future.

## Conclusions

The mean visual acuity of the SRD group was worse than that of the CME group, but the macular sensitivity of the two groups did not differ significantly. This suggests that SRD itself may be related to a decrease of visual acuity together with CME, because nearly all of the SRD patients also had CME. However, SRD had no influence on macular sensitivity because there was no difference of sensitivity between the SRD and CME groups.

## Competing interests

The authors declare that they have no competing interests.

## Authors' contributions

HN, and HF were involved in the design and conduct of the study. Collection and management of the data were done by HN, and KS, while analysis and interpretation of the data were performed by HN, HF, TM, and KS. Preparation of the first draft of the manuscript was done by HN, and review and approval of the manuscript was performed by HF and TM. All authors read and approved the final manuscript.

## Pre-publication history

The pre-publication history for this paper can be accessed here:

http://www.biomedcentral.com/1471-2415/11/29/prepub
